# Communication Skills of Medical Interpreters: A Qualitative Explanatory Study of Healthcare Professionals’ and Medical Interpreters’ Perceptions

**DOI:** 10.3390/healthcare12202073

**Published:** 2024-10-17

**Authors:** Naoko Ono, Jinghua Yang

**Affiliations:** 1 Faculty of International Liberal Arts, Juntendo University, Tokyo 113-8421, Japan; 2 Graduate School of Medicine, Juntendo University, Tokyo 113-8421, Japan

**Keywords:** medical interpreters, medical professionals, communication skills

## Abstract

**Background/Objectives**: Medical interpreters support communication between medical professionals and foreign patients. However, the communication skills required of medical interpreters in the field are currently unclear. The purpose of this study was to investigate what medical professionals and medical interpreters consider to be most important communication skills of medical interpreters, and whether there are differences in perceptions between medical professionals and medical interpreters. **Methods**: From December 2023 to February 2024, we interviewed 10 medical professionals and medical interpreters (five medical professionals and five medical interpreters) working throughout Japan and in the United States. The narratives collected in the interviews were analyzed and evaluated in a conceptual framework. **Results**: Healthcare professionals and medical interpreters exhibited differences in perceptions regarding the most important communication skills for medical interpreters. The narratives of medical professionals and medical interpreters regarding medical interpreters’ communication skills were divided into a 2 × 2 grid consisting of four quadrants with two axes (intervention and perspective). Regarding the communication skills of medical interpreters, medical professionals were primarily concerned with the transmission of medical information and felt that it was necessary for interpreters to understand medical professionals’ perspectives and what they wanted to say in order to convey it accurately. In contrast, medical interpreters emphasized the importance of empathy and communication skills for respecting the other person and understanding the other person’s opinion and perspective. Additionally, to understand the patient’s perspective, medical interpreters reported that it was important to understand the cultural background of the patient and to maintain good relationships with all of the people around the patient. **Conclusions**: We explored the communication skills of medical interpreters and the characteristics of the perceptions and narratives of medical professionals and medical interpreters. Concepts regarding the communication skills of medical interpreters were divided into two axes (intervention and perspective) and presented as a 2 × 2 grid with four quadrants. The results of our study suggested that medical interpreters should actively intervene in interpretation situations involving foreign patients.

## 1. Introduction

Healthcare and patient communication is at the heart of medical practice. However, communication with foreign patients is difficult because of language barriers. The Basic Survey on Foreign Residents in FY2022 revealed that over half of foreign residents (52.8%) required language assistance when seeking medical care [1]. A previous study suggested that if communication between medical professionals and patients is not accurate, it may be dangerous not only for patients but also for medical professionals [2]. Additionally, it has been reported that difficulties in verbal communication can interfere with communication between medical professionals and patients, such as medication guidance [3].

In today’s increasingly globalized Japan, there is a rising demand for medical interpreters to accommodate the growing number of foreign patients. While the number of foreign visitors to Japan significantly declined because of the COVID-19 pandemic, international travel has resumed under “With/Post-Corona” measures, leading to a renewed increase in foreign visitors. At the same time, the number of foreign residents is also rising, and the influx of foreign patients at medical institutions in Japan is expected to grow.

To address this need, the International Society of Clinical Medicine in Japan has established a certification examination for medical interpreters. As of April 2024, 423 medical interpreters had been certified by the society [4]. However, there remains a significant gap between the number of certified interpreters and the demand from medical institutions. A 2016 survey by Hamai et al. [5], which assessed the needs for medical interpreters at local government hospitals across Japan, revealed that 75.7% of small hospitals and 92.3% of large hospitals would use medical interpreters if they were covered by medical fees. A 2023 Ministry of Health, Labor, and Welfare (MHLW) survey [6] showed that of 335 secondary medical care areas (regions comprising multiple municipalities providing general inpatient treatment, including emergency care), 139 (41.5%) areas had hospitals with medical interpreters, 212 (63.3%) areas had hospitals offering telephone interpreting services, and 108 (32.2%) areas had hospitals utilizing video interpretation.

Medical interpreters work in various capacities, including those directly employed by medical institutions, those employed by private businesses (e.g., corporations), and those affiliated with nonprofit organizations (e.g., government agencies). Whether working in person or online, medical interpreters are expected to collaborate with healthcare professionals in hospitals. However, not all medical interpreters are certified by the International Clinical Society, and Japan currently lacks a unified interpreter registration system. As a result, many hospitals rely on untrained volunteers and non-governmental organizations to fill the gap.

Medical interpreters support communication between medical professionals and foreign patients. Communication through the intervention of untrained interpreters can lead not only to poor language comprehension but also to dysfunction of team medicine, cultural conflicts and on-site confusion, increased human costs for hospital operations because of complaints against interpreters, and medical lawsuits caused by mistranslations [7]. However, some previous studies reported that the presence of trained medical interpreters increased the use of health services by patients with language or communication difficulties [8]. Lion et al. [9] reported that the use of professional interpreters was linked to factors such as communication length and content. Their use was more frequent during medical history interviews and less common in high-risk activities such as medication administration. Thus, the role of trained professional medical interpreters is important for providing a bridge of communication in medical care.

Regarding the communication skills of medical interpreters, the MHLW has published a list of types of communication: intercultural communication, interpersonal communication, how to interact with patients, relationships between patients and healthcare professionals, health, medical care, and cultural and social differences. Additionally, five types of cultural mediation have been defined by the MHLW [10]. In fields other than medical interpreting, there are many measures of communication skills [10]. For example, Fujimoto and Daibo developed the Encode, Decode, Control, and Regulate Scale (ENDCORES) [11]. In this model, encode refers to expressiveness and self-assertion, decode refers to the decoding and acceptance of others, control refers to self-control, and regulation refers to relational adjustment [12]. Thus, this communication skills scale measures six categories: self-control, expressiveness, decipherment, assertiveness, acceptance of others, and relational adjustment. These communication skills may also apply to the evaluation of communication skills of medical interpreters. However, previous studies have not verified what kinds of communication skills are required of medical interpreters.

### Objectives

The current study was guided by two research questions:

1. What do medical professionals and medical interpreters consider to be the most important communication skills of medical interpreters?
2. What are the differences in perceptions between the two parties?

## 2. Materials and Methods

### 2.1. Study Duration, Location, and Participants

The interview planning, protocol design, ethics approval, recruitment, consent process, interviews, transcriptions, analysis, and write up were conducted between October 2022 and April 2024. Participants were selected from among medical professionals who resided in Japan or the United States, and who used medical interpretation services or had experience in medical interpretation services. Japan, as a primary cultural context for patients, was central to the study. To explore the communication skills of medical interpreters working with the Japanese language in Japan, we focused on interpreters between English and Japanese as well as Chinese and Japanese. Ten individuals (one man and nine women) were selected; they comprised five medical professionals (two doctors and three nurses) and five medical interpreters.

Participants were chosen from the authors’ network of acquaintances with experience in medical interpretation. Two of these participants, although selected from Japan, were residing in the United States at the time of the interviews. The study involved four medical institutions. While most participants discussed medical interpretation within Japan, one individual (G) shared their experiences related to medical interpretation in the United States.

### 2.2. Recruitment Methods and Approaches

Regarding the recruitment method, participants were selected from medical interpreters known to the authors, with an emphasis on balancing experience levels and representation from various medical professions. Because English and Chinese are the most commonly taught languages for medical interpretation in Japan, interpreters fluent in these languages were chosen for the study. Two of the selected participants were originally from Japan but were residing in the United States at the time of the study. The interviews lasted between 40 and 60 min, and all sessions were recorded, transcribed, and analyzed. Potential participants were contacted, and the purpose of the study was explained to them through a written explanation. Written consent was obtained prior to their participation.

Employers of the medical interpreters included medical institutions, private interpretation agencies, travel agencies, and nonprofit organizations. Some participants worked as freelance medical interpreters contracted by multiple agencies, while others were mainly contracted by nonprofit organizations. Additionally, some participants were employed, either regularly or irregularly, as medical professionals and provided medical interpretation as part of their regular duties. Because of overlapping roles, such as being registered with both private agencies and non-profit organizations, it was difficult to classify them into distinct categories.

### 2.3. Data Collection

An online interview was conducted in Japanese via Zoom. Additional interviews were conducted as needed. The content of each interview was recorded with the participant’s consent, and transcripts were prepared.

In the interviews, four main questions were asked: “What kind of communication is required of medical interpreters in the field?”, “What are some examples of problems that arise when medical interpreters lack communication skills?”, ”What do you think are the most important communication skills of a medical interpreter and why?”, and “What do you want from a medical interpreter’s communication training?”. Semi-structured interviews were conducted on the basis of the interview guide, and participants were encouraged to speak freely. The communication skills included the six items [11] of the simplified version of ENDCORES and the five items of communication skills of medical interpreters reported by the MHLW [10] (Table 1). Each interview lasted 40–60 min.

### 2.4. Data Analysis

The recording of each interview was transcribed after the interview. The transcripts were analyzed using the “Coding and Theorizing Steps” method established by Otani [13], which is Japan’s first internationally recognized qualitative analysis method, known as SCAT. This process involves segmenting the text and entering it into a specialized form for analysis, starting with verbatim transcription.

The method involves a five-step coding process to identify themes and structures, interweave the themes and structures to develop a storyline, and finally provide a theory [14,15,16,17,18,19]. We chose this approach to test the theory by guaranteeing an explicit analysis process and an opportunity to critique and show falsifiability.

The analysis was performed according to the following procedure:

(1) Identify Key Words and Phrases
  - Read through a line of text.
  - Write down any words or phrases that stand out, especially those related to the research topic, or that raise questions or seem unclear.

(2) Paraphrase Key Words and Phrases
  - Rephrase or summarize the words/phrases written in Step 1.
  - The goal is to generalize these specific instances into broader concepts that apply to the research focus.

(3) Identify Out-of-Text Concepts
  - Write down any external concepts, ideas, or theories that help explain or expand on the paraphrased ideas from Step 2.
  - This could involve looking at causes, effects, comparisons, or characteristics that were not explicitly mentioned in the text but provide additional insight.

(4) Determine a Theme
  - After going through Steps 1–3, decide on a unifying “theme” that ties the concepts together.
  - If a theme is not clear, write down common phrases or ideas that come up frequently to help identify one.

(5) Reflect on Questions and Issues
  - List any questions or issues that came up during the analysis.
  - Compare these data with other parts of the same interview or other interviews to find patterns or discrepancies.
  - Consider if follow-up interviews or further literature research are needed to explore these points in more detail.

Based on the identified “themes”, a storyline was constructed. The coders described the underlying meaning and significance of the events in the data, weaving together the themes identified in Step 4. They expanded the storyline by comparing and connecting smaller storylines from different parts of the data. After creating the storyline, the researchers made a theoretical statement. They wrote the statement as a knowledge or theory, not as an analyst’s idea. Finally, the authors expressed the theory using the language and terms found within the storyline itself.

By analyzing the results of these interviews, we constructed and generated theoretical writing and explored the perceptions of medical professionals and medical interpreters regarding the communication of medical interpreters.

In addition, after constructing the storyline, the text was further refined. Concepts were extracted from the storyline, summarized, and organized into a framework. This narrative framework served as a conceptual tool to clarify and generalize the nature of the problem [20].

The four coders were trained in the analysis method and then conducted their analyses independently. To ensure intercoder reliability, the results were compared, and any differences were discussed until consensus was reached. Discussions among the coders were also held to develop and stabilize the coding tree.

### 2.5. Ethical Consideration

This study was conducted in accordance with the Declaration of Helsinki and was approved by the Research Ethics Review Committee of the School of International Liberal Arts Studies, Juntendo University (approval date: 6 September 2023, approval number: 0022). Informed consent was obtained from all participants involved in the study.

## 3. Results

In total, ten interviews (five medical professionals and five medical interpreters) were conducted (Table 2). Of the ten participants, nine were female and one was male. The participants ranged in age from their 20s to their 70s. Of the participants, seven were Japanese and three were Chinese. The minimum number of years of experience as a medical interpreter/medical professional was 4 years, and the maximum was 27 years. The sample included four freelance workers, two part-time workers, and four full-time workers. The respondents included three native Chinese-speakers and seven native Japanese-speakers. Healthcare providers (except participant I) acted as interpreters too. The sample included one telephone interpreter and nine face-to-face interpreters. Participant G had experience as an English–Japanese interpreter in the U.S. Forces in Japan. So, participant G worked with the Japanese language in Japan, but the language in which the healthcare consultation was performed at that time was English. The important attributes for the communication skill of medical interpreters are 4b-5, 6, 8, 10 (two for each), followed by 4b-3, 6 (one for each).

The results of analyzing the interview and conducting a theoretical description are presented with examples of utterances for each question.

In response to the question of what kind of communication is required for medical interpreters in the field, medical professionals reported that interpreters should adjust their communication skills on the basis of understanding the patient’s cultural background, in addition to accurately conveying medical information through interpretation. From the interview of (Example 1), it is suggested that medical interpreters are expected to accurately convey the patient’s emotions, nuances, and environmental factors to the medical staff. The importance of nonverbal communication was emphasized by a respondent (Example 2), and from the interaction with the respondent F, we understood that it would contribute to building a relationship of trust between patients and healthcare providers as a bridge to enable communication beyond words, paying attention to the tone of voice and the manner of speaking.

<Example 1. Respondent I, Nurse/Midwife>

*Of course, I want medical interpreters to be concerned about their patients, such as their physical condition, and so on, but I think the most important thing is that the interpreter prioritizes the patient, and that they play a role in accurately bridging the gap between what we have communicated and what the patient wants to convey.*

<Example 2. Respondent F, Nurse>

*I’d like interpreters to pay attention to the patient’s facial expressions, the environment of the hospital room, and things that may be unsuitable for the patient. As a person who stands on the patient’s side, not from the perspective of a medical professional, I am grateful that interpreters can see and convey the inconveniences of hospitals and other aspects that are different from daily life.*

From the interaction with the respondent B, it was inferred that medical interpreters, like medical professionals, were expected to go beyond simply interpreting words, to promote understanding and cooperation among all participants toward their treatment goals through cultural sensitivity and interpersonal relationships (Example 3). Regarding the importance of nonverbal communication, respondents emphasized the importance of medical interpreters deepening trust and understanding by building rapport through greetings and self-introductions (Example 3), expressing professionalism (Example 4) (in their dress, and formal language), observing the situation, and applying appropriate communication strategies according to the situation.

<Example 3. Respondent B>

*For example, no matter whether you are a staff member and an interpreter at a hospital, or if you are an interpreter who comes from outside or is dispatched, when you are introduced to a patient, you should first talk to them and introduce yourself. At that time, when a social worker introduces me, I take time to speak to the social worker and the office staff to introduce myself. When you enter the examination room, I think it is important to introduce yourself, to clarify your role, and to greet the doctor.*

<Example 4. Respondent E>

*I think it’s important to dress properly and look professional, even if it’s just appearance, so I always wear a jacket. Also, I always talk in formal language.*

Regarding examples of problems that arise when medical interpreters lack communication skills, medical professionals described examples in which the lack of interpreter skills can lead to a risk of misunderstanding and misdiagnosis because of miscommunication or omission of important medical information, the potential for patient misunderstanding and anxiety, and the direct impact on patients’ health (Example 5). In addition, respondents (Example 6) pointed out that the interpreter directly answering the patient’s questions during the consultation can lead to making medical judgments despite the lack of specialized medical knowledge, which can cause serious problems.

<Example 5. Respondent G, Doctor>

*I think it’s very common for things to happen when something that you thought was conveyed through an interpreter is not being conveyed. For example, sometimes the patient does not understand what he or she is going to do next, or doesn’t understand the medical procedure.*

<Example 6. Respondent I, Nurse/Midwife>

*Medical interpreters have received certain training to be a medical interpreter, haven’t they? In that case, I guess that there is a certain amount of medical knowledge that they gain from their own experience. However, medical interpreters should not answer questions from medical professionals on behalf of patients, or* vice versa*. If that happens, medical professionals cannot be held responsible for the information they provide. If the content is different, I think it can cause problems.*

Some respondents suggested that an interpreter’s lack of communication skills can lead to a poor understanding of the patient’s health condition and treatment plan, which can affect the effectiveness and safety of treatment. This was inferred from the interaction with the respondent C (Example 7). In particular, it was pointed out that understanding and communicating cultural nuances is important for building trust between patients and providers, and that understanding and adaptation to cultural differences can lead to effective communication with patients and reduce problems. One respondent (Example 8) also gave examples of the transparency of communication and its importance. In particular, it was reported that accurately conveying the meaning and context hidden behind words is essential for building trust and sharing information between patients and healthcare providers.

<Example 7. Respondent C>

*It was an explanation of the endoscopic examination, and the physician said that he was explaining it. The physician and I both understood that it was an explanation of the anesthesiology for the examination the following day. I was interpreting for a long time, and it was only at the end that it became clear that the patient seemed to think that it was an explanation of the anesthesia for the main examination or the main surgery. But the information I was interpreting was not about the exam for the main surgery, but an explanation of anesthesia for the examination the next day.*

<Example 8. Respondent A>

*There are cultural metaphors in the words used in each language. One day, a Chinese patient said, “Yesterday, my grandma came. Now my stomach hurts”. The phrase refers to periods. Among Chinese women, and even men, everyone understands this metaphor. But when my Japanese colleague heard that, he said, “Are your relatives coming to Japan?”*

Regarding respondents’ perceptions (Example 9) of the most important communication skill for medical interpreters and the reason, the item most frequently selected by medical professionals was the ability to understand the relationship and communication between patients and medical professionals (item 4B-8). To improve the quality of communication between patients and providers, it is necessary to understand what healthcare providers want to ask patients. The information needs to be organized, summarized, and adjusted accordingly. This process requires the skills of a medical interpreter to balance what the patient wants to say with the information the healthcare provider needs to obtain, while also meeting the needs of both parties. This includes the ability to faithfully deliver the patient’s words while also providing the information in the form that the healthcare provider requires. It is also important to have an accurate understanding and self-awareness of the role of a medical interpreter, emphasizing the avoidance of actions that go beyond the role of a healthcare provider.

<Example 9. Respondent F, Nurse>

*There is a big difference between what we want to hear and what patients want to talk about. We want to hear about the patient’s illness and condition. What patients often want to talk about is what they are having trouble with because of their illness, the changes they have made in their daily lives, and other factors that are not related to medical care. Of course, there are some positive aspects to talking about these things, but if they talk about them too much, you may not be able to hear it all, or you may not be able to get the information you need, even though there is something important you want to ask. If medical staff understand what they are trying to ask, we can ask the patient open questions using relatively mild language; however, if medical interpreters are able to narrow it down little by little, it is easier for the patient to understand, and the medical staff will be able to ask for the information they need.*

In addition, item 4b-3 (correctly reading the thoughts and feelings of the other person) was selected by medical professionals as an important communication skill for medical interpreters. From the interaction with respondent H (Example 10), we suggested that medical interpreters can understand not only the patient’s words but also the intentions, emotions, and anxieties behind them, and communicate them to the healthcare professional, encouraging them to propose a more personalized treatment plan and actively participate in the patient’s treatment. At the same time, it is necessary for interpreters to convey information from healthcare professionals in a language that is easy for patients to understand, reducing their anxiety and increasing their sense of satisfaction in their treatment choices. Respondents also emphasized the need for medical interpreters to go beyond language translation to bridge differences in cultural backgrounds, values, and expectations.

<Example 10. Respondent H, Nurse>

*If you don’t understand correctly why medical professionals want to do something, and what they really want, you probably won’t be able to convey it accurately to the patients. Chinese physicians tend to give direct orders to patients. In contrast, physicians in Japan offer patients options. Even though they really want the patients to do something, they often ask for the patient’s consent. I think it’s important to understand Japanese physicians’ intentions properly, and to convey these to the patient. The patient can then say whether they will follow the doctor’s advice, or whether they will get treatment.*

In particular, professionalism, emotional control, and perseverance were reported to be essential qualities for medical interpreters to perform their work effectively. In addition, respondents (Example 11) reported that medical interpreters should possess a professional attitude that includes respect for patients and healthcare professionals, empathy regarding patients’ emotions, and the ability to remain calm in any situation.

<Example 11. Respondent G, Doctor>

*It is common for medical professionals to find that communicating with dementia patients is frustrating, and I think that interpreters are no exception. I’ve had patients who have a hard time expressing what they’re trying to say, who don’t give a response no matter how many times they are asked, or who keep talking endlessly about things that have nothing to do with what the interpreter has heard, over and over again. It’s a common occurrence with older people. I think anger management is important in its own way.*

The most common items selected by medical interpreters as the most important communication skills for medical interpreters were respecting the other person and understanding the other person’s opinion and position (item 4b-5), working with the relationships around you, and maintaining flexibility (item 4b-6). Respondents (Examples 12, 13) reported that these two items are essential for medical interpreters to achieve their goals of choosing the correct words, communicating intentions accurately, and ensuring respect and fairness for patients. Respondent E (Example 14) explained the importance of interpreters aligning with and contributing to healthcare providers’ goals, in particular, with rapport building.

<Example 12. Respondent B>

*You need to understand the other person’s opinion. Even if the physician says something harsh, you need to understand what their intention is. For example, a physician might say that the patient is not taking their medication properly and that this needs to be translated in a way that conveys their point strongly; in such a case, it is not acceptable to downgrade the words and weaken the message. After all, the other person’s intentions, the physician’s position, and the physician’s opinion may have particular reasons behind them; so if you need to confirm the intention, you need to confirm it, and then convey it without diluting or strengthening it yourself.*

<Example 13. Respondent C>

*If you don’t understand what the other person really wants to say, whether it’s a doctor or a patient, you can’t say that it’s an accurate interpretation. Accuracy leads to the most direct communication.*

<Example 14. Respondent E>

*When you have a good relationship with patients and medical professionals, everything goes well. When a good relationship can be built between the patient and the doctor, and between the medical staff and the patient, then a relationship of trust will naturally develop. If you can do that, the treatment will work well. The goal is to treat the patient and help them get better, so the interpreters should work toward that goal. As an interpreter, I try to emphasize the positive words from the doctor to the patient, such as “take care of yourself” or “let’s get better”. I would like to say that with a little excitement.*

Regarding item 4a-10 (understanding cultural and social differences, such as lifestyle habits and values, as well as religious views and beliefs related to health, medical care, and communication), medical interpreters feel that overcoming cultural diversity and language barriers are important for improving access and the quality of healthcare (Example 15). However, if the interpreter’s intervention is not performed properly, it can lead to communication confusion and wasted time.

<Example 15. Respondent A>

*In my experience as an interpreter, I have encountered quite a few Chinese patients who have taken herbal medicines or other medicines without permission. I think that this is not considered to be acceptable in Japan. I can understand that patients might take aspirin without permission, but Japanese physicians do not understand it. I think it is important for interpreters to understand differences in such values regarding health.*

Regarding the desired communication training for medical interpreters, a medical professional (Example 16) mentioned that nursing students often use photographs to develop skills to deal with the complexity and unpredictability of healthcare. Respondents reported that it is desirable for interpreters to have training in understanding the medical environment, the importance of nonverbal communication, and how to respond appropriately to the situation. Training should include an accurate understanding and use of medical terminology, as well as the development of the ability to appropriately grasp and communicate the needs of patients and healthcare professionals. Additionally, respondents (Example 17) reported that cultural sensitivity is also important, recognizing that communicating effectively with people from different cultural backgrounds requires understanding of the customs and taboos of a culture. Respondents described the need to strengthen the art of intercultural communication through practical role-play and case studies.

<Example 16. Respondent F, Nurse>

*When I was a nursing student, I was shown pictures in class and asked what I would do in such a situation. I answered, “I think this is what I would do”, and I was told at the beginning that there was no right or wrong answer. Then I was asked to explain my response. I think it was a good learning experience to be able to explain what would happen if a nurse was there, or if a particular kind of doctor was there. Students were required to explain various patterns of flexible behavior. I think I learned a lot by being taught about various patterns and examples.*

<Example 17. Respondent G, Doctor>

*In France, it seems that pointing out skin color or hair style is acceptable, but in the United States, it seems to be taboo. In Japan, they don’t care about that, so they don’t know.*

Medical professionals mentioned that interpreters need to function as part of the healthcare team and to follow specific protocols and ethics in a medical setting. This includes promoting appropriate self-introduction, confidentiality observance, and effective communication between healthcare providers and patients. Practice through role-play allows medical interpreters to simulate various scenarios that they may face in a real-life medical setting (Example 18). This allows them to develop practical skills such as translating medical terminology, coordinating communication between cultures, and responding quickly in an emergency.

<Example 18. Respondent J, Doctor>

*It is desirable for training to develop the ability to deal with problems as they arise. In terms of how to deal with misunderstandings or situations in which medical knowledge is not translated well, I think it is good to get together and practice with case studies and role-play.*

From the interaction with respondent D (Example 20) and respondent B (Example 21), we inferred that, for interpreters to function effectively, they need to improve their general communication skills and cultural awareness. This requires extensive training (Examples 21), including intercultural communication, non-verbal communication, and emotional expression. This is essential to avoid misunderstandings between patients and healthcare providers and to provide appropriate medical care. Listening skills include the ability to accurately understand and respond appropriately to information from patients and healthcare providers. This forms the basis for building trust and effective communication.

Responding to emotional challenges effectively requires developing an understanding of how medical interpreters deal with emotional situations they may face, especially when communicating difficult medical information (Example 19).

<Example 19. Respondent C>

*There’s abuse, there’s violence, there’s sexual abuse, there’s all kinds of abuse, right? That’s why it’s important to understand your own mentality when dealing with that kind of thing. It’s also very important to know what you’re going to do when you face such a situation. What I want the training program to include is developing an understanding of what kind of person you are when something happens. I think that’s very important. For example, there may be some people who hear a very painful story and feel really sorry, and they can’t get it out of their head, or they want to get in touch with the person affected. It’s important that you can understand how your mind works when you encounter a situation like that. I think that it can make a person a little more objective. I think it would be good for this to be included in the training, to help people understand themselves.*

In addition, respondents reported that it is useful for interpreters to know about the content of the training received by medical professionals who are in contact with patients. Respondents (Example 22) described the importance of medical interpreters being able to understand the intentions of medical professionals and to understand the context. Medical professionals reported that it would be useful to acquire knowledge that is not tailored to medical interpreters, such as an understanding of the things that doctors, pharmacists, and nurses have learned.

<Example 20. Respondent D>

*The Japan Automobile Federation (JAF) has a training program called Hazard Prediction Training [21]. The JAF training program is available on YouTube, but there is also a version with a single photo. JAF’s magazine is published every month, and contains a quiz that asks the reader to identify what a driver needs to look out for. For example, you might see a photo of a child on the street. There’s a glimpse of the ball. There’s always a kid running after the ball, and that’s what the driver has to be aware of. This type of training program could be useful for medical interpreters.*

<Example 21. Respondent B>

*A simulation or a mock practice may be useful for people who have never performed interpretation in a hospital setting. It could start with a greeting and then explain a little bit about themselves. It could provide a run-through, including advice about which words are important to include.*

<Example 22. Respondent E>

*A training program in which medical professionals learn how to interact with patients. When an interpreter goes into the field without any medical knowledge, they may have difficulty understanding the medical professional’s questions. If you know the intention of the medical professional asking the questions, you don’t have to add any extra words.*

### 3.1. Recognition of the Characteristics of Medical Interpreters’ Communication Skills (2 Axes)

**Figure 1:**
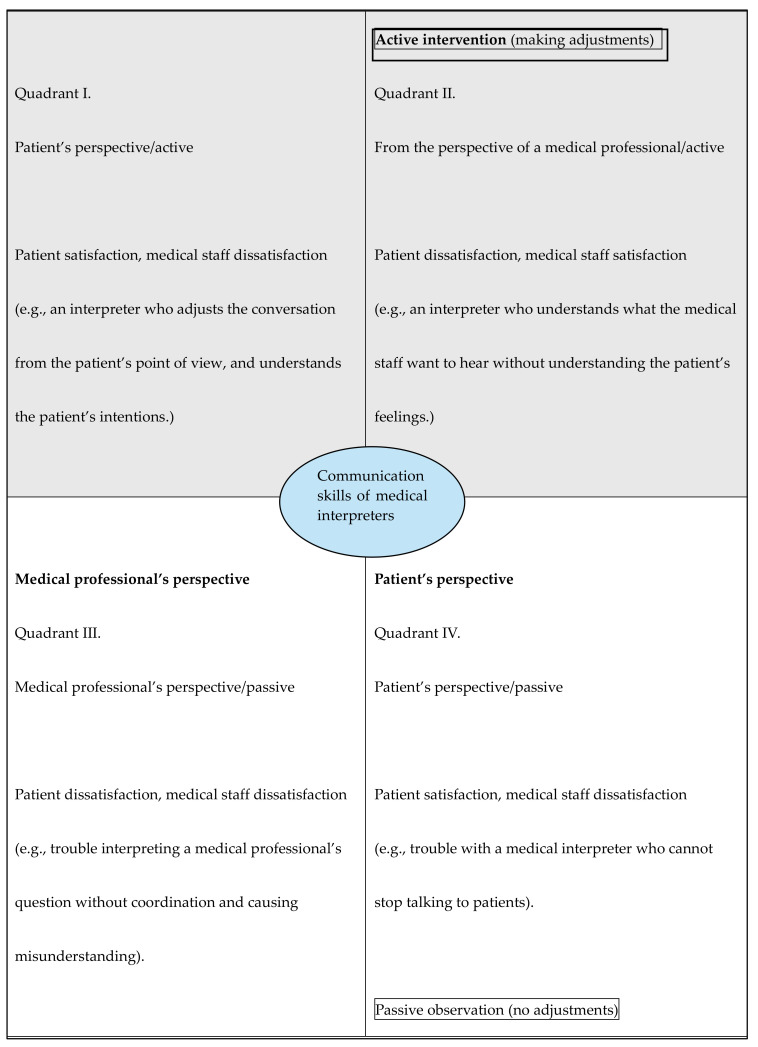
Conceptual diagram of the communication skills of medical interpreters.

The communication skills of medical interpreters as described by medical professionals and medical interpreters were classified according to “intervention” and “point of view” in a four-quadrant 2 × 2 grid with two axes (Figure 1).

The content was based on the axes of “intervention” and “medical professional/patient’s perspective”, consisting of “active (intervention)” and “passive (observation)” categories. “Medical professional/patient’s perspective” refers to whether the communication is satisfactory from the perspective of each position: satisfaction/dissatisfaction, and typical examples of this are shown in parentheses.

Regarding passive observation, it was considered that the highly developed communication skills of medical interpreters included the ability to actively intervene as necessary and to adjust their communication in consideration of the positions of both the medical staff and the patient.

The textbook definition of a medical interpreter’s role is to “add or subtract nothing”; i.e., they are expected to convey information exactly as it is, acting like a conduit without influencing the interaction. However, as Wadensjö [22] argued, and as shown in Figure 1, interpreters do more than simply relay verbal information. They actively use their communication skills to engage in the interaction, making them participants in the exchange rather than passive conduits.

### 3.2. Perception of the Content and Structure of the Communicative Competence of Medical Interpreters: Organization of Two Axes and Four Quadrants

The created storylines based on interviewees’ responses were analyzed and divided into four quadrants according to two axes: the medical professional’s perspective and the patient’s perspective and passive observation and active intervention. The categories were assigned to the quadrants as follows (Figure 1):

1. Quadrant I:
  Patient’s perspective/active intervention
2. Quadrant II:
  Medical professional’s perspective/active intervention
3. Quadrant III:
  Medical professional’s perspective/passive observation
4. Quadrant IV:
  Passive observation/subjective structure.

The first quadrant represents an active attempt to be close to the patient and to resolve their doubts. In this situation, the patient is satisfied, but the medical staff are dissatisfied because the consultation time is extended. The second quadrant included the interpreter’s perception of what the medical staff wanted to hear without understanding the patient’s feelings. Although the information that the medical professional wants to know can be obtained, the patient may feel that his or her feelings have been ignored, and they may be dissatisfied. In the third quadrant, information is collected from the perspective of medical professionals, but they cannot obtain the information because interpreters did not adjust the patient’s speech, and the patient is unable to resolve their doubts, which makes both parties dissatisfied. Quadrant IV represents a situation in which speech is interpreted and not coordinated as desired from the patient’s point of view. The patient can say what he or she wants to say, but the information is not organized for the recipient, leaving the medical staff dissatisfied. According to the analysis of the interview results and this 2 × 2 grid, the ideal medical interpreter’s communication skills are considered to be active, falling somewhere between Quadrant I and II.

## 4. Discussion

The current study had two main objectives. First, we aimed to identify what medical practitioners and medical interpreters consider to be the most important communication skills of medical interpreters. Second, we sought to understand differences between the perspectives of medical professionals and patients.

In response to the first question, participants identified the most important communication skills for medical interpreters. The top skills, each mentioned twice, were 4b-5 (respect others and understand their opinions and positions), 4b-6 (work with relationships and maintain flexibility), 4b-8 (understand the relationship and communication between patients and healthcare professionals), and 4b-10 (understand cultural and social differences, including lifestyle habits, values, religious views, and ideas related to health, medical care, and communication). Additionally, 4b-1 (take control of your emotions and behaviors) and 4b-3 (correctly understand the thoughts and feelings the other person wants to convey) were each mentioned once. Also, 4b-5 (respect others and understand their opinions and positions), 4b-6 (work with the relationships around you and maintain flexibility), 4b-8 (understand the relationship and communication between patients and healthcare professionals), and 4b-10 (understand cultural and social differences such as lifestyle habits and values, religious views and ideas related to health, medical care, and communication) contain understanding and relationship commonly.

Bernardi and Gnani [23] noted that medical interpreters and cultural mediators, who often play overlapping roles, are particularly important in breaking down language barriers.

La Vonne et al. [24] stated that being able to understand a doctor’s explanation has a positive impact on patient satisfaction, while a lack of understanding leads to a decrease in patient satisfaction. The results of the present study are consistent with Street et al.’s assertion that verbal communication between patients and medical professionals in clinical settings is highly sensitive and requires a high degree of sensitivity [25].

Medical professionals and medical interpreters required medical interpreters to understand what medical professionals wanted to say to the patient and to accurately convey medical information to the patient. Additionally, to enable the patient to understand, listening skills and an ability to understand the patient’s situation and emotions were reported to be required. Respondents also reported that it is important to understand the patient’s culture and background, and to maintain a good relationship with the person concerned.

The ideal medical interpreter’s communication skills are considered to be active, meaning that medical interpreters required to understand others and intervene relationships between patients and other medical staff actively. This is consistent with previous research that medical interpreters are required to play the role of cultural mediators as well as linguistic mediators [26].

Regarding the second question, when we compared the interview responses of medical professionals and medical interpreters regarding the communication skills required by medical interpreters in the field, they agreed that medical interpretation involves more than verbal interpretation, and also involves cultural sensitivity, nonverbal communication, and communicative coordination.

An important difference was that medical professionals considered medical interpreters to have the perspective of outsiders who were not medical professionals but were aware of the patient’s thoughts and desires, while medical interpreters did not express an awareness of their position as “outsiders” in relation to medical professionals. On the contrary, interpreters reported that, by observing the situation and adopting a communication strategy according to the individual, they aimed to build trust as members of the medical team. In a previous study of minority medical students, they actively excluded their personal identities as outsiders in the dominant culture of medicine [27]. The results of this study are similar to the attitude of medical interpreters who seek to understand medical professionals and patients by putting aside their identities in order to adapt to the clinical setting and medical culture. However, in this study, the medical practitioners expected medical interpreters to be seen as outsiders, which was different from the claims of previous studies. Medical interpreters were expected to adapt to clinical situations without discomfort and to detect the patient’s wishes from the standpoint of non-medical professionals as necessary.

Comparison of the interviews between medical professionals and medical interpreters revealed that medical professionals cited problems caused by miscommunication of medical information and problems that undermined the relationship of trust between patients and medical professionals caused by misunderstanding of cultural differences and miscommunication. Cultural factors included not only the cultural context of the foreign patients’ country but also the culture of the hospital.

In contrast, medical interpreters emphasized problems caused by cultural and healthcare system background factors that medical professionals did not emphasize.

These study results reveal two important cultural aspects that need to be considered separately. The first aspect concerns the culture within hospitals. Hospitals, aside from exceptions such as routine medical examinations, are primarily facilities for treating the sick. The healthcare professionals working in hospitals receive specialized education, pass national exams, and undergo clinical training. A hospital is a unique environment where professionals gather, almost like a foreign country compared with the outside world. Each healthcare professional has a distinct role (e.g., doctors determine and direct treatment), and they understand not only their own roles but also those of their colleagues, all working toward the common goal of healing. Speech within this setting is part of action, with clear intentions behind it. Healthcare professionals expect medical interpreters to grasp not only the literal meaning of their statements but also the intentions behind them. Even if interpreters do not fully understand the context, they are expected to provide accurate interpretation that conveys the intended message. Interpreters who successfully aligned their interpretation with the healthcare professionals’ intentions were seen as providing greater satisfaction.

The second cultural aspect relates to the culture of each country. In this study, significant data were obtained, particularly on Chinese culture. Medical interpreters must understand not only the language but also the unique jargon, the relationship dynamics between medical professionals and patients, and the communication style prevalent in the target country. They adjust their translations to align with the cultural context of the target language, using facial expressions and tone to bridge gaps. If they were to adhere strictly to a “nothing added or subtracted” approach, it could result in misunderstandings or incomplete information. Medical interpreters who are familiar with both cultures understand that rigid literal translation could be disrespectful, and they work to coordinate communication effectively. Köksala and Yürük [28] emphasized that interpreters need to enhance their sensitivity not only to linguistic differences but also to cultural ones to minimize misunderstandings and ensure successful intercultural communication. For example, in Chinese culture, medical professionals are seen as authoritative figures, and patients are more likely to follow their recommendations without question. In this context, a “patient-centered approach” that does not provide patients with enough background information can negatively affect the relationship between healthcare providers and patients. Pokorn and Južnič [29] found, through a textual comparison for community interpreters and intercultural mediators, that while the Code of Ethics for Community Interpreters tends to emphasize impartiality, the role of intercultural mediators often involves a greater focus on advocacy. This study also highlights that medical interpreters not only provide neutral, impartial interpretation but also act as intercultural mediators, adjusting communication as necessary to facilitate understanding.

When comparing the interview responses of medical professionals and medical interpreters regarding the most important communication ability of medical interpreters and why, we found that there were some differences. Medical professionals felt that to convey information accurately, it was necessary to understand the perspective of medical professionals and what they really wanted to say. In addition, patience, self-control, and understanding of the patient’s situation and emotions were required to enable the patient’s understanding.

Medical interpreters, in contrast, believed that respecting and understanding the other person’s opinion and position was essential for achieving the goal of accurately communicating the intentions of the medical practitioner and the patient, and ensuring respect and fairness for the patient. To that end, medical interpreters emphasized empathy and communication skills. In addition, to understand the patient’s position, it was important to understand the cultural context and background. Additionally, medical interpreters reported that it was important to maintain good relationships with all of the people around the patient, in addition to the medical personnel and the patient themselves. Medical interpreters were aware that in order for foreign patients to be satisfied with their medical care, it was necessary not only to accurately interpret technical terms, but also to support patients to build relationships of trust and build good relationships with those around them. This is consistent with past research that it is important to build a relationship of trust with patients and to support them when communicating with foreign patients [30,31,32,33].

Comparing the interviews between medical professionals and medical interpreters regarding their expectations for communication training for medical interpreters revealed that both medical professionals and interpreters suggested role-playing and case studies as training methods to develop practical skills to accurately and flexibly respond to actual events in the medical field in a hands-on manner. In contrast, medical interpreters made practical suggestions, such as the use of the type of simulation training used to prevent car accidents, and also suggested that training to improve response skills, such as listening attentively and processing one’s own emotions, may be useful. In addition, medical interpreters reported that they were not aware that medical professionals were trained to interact with patients, and felt that medical professionals needed to have these skills. It is also considered necessary to train medical interpreters on their own mental care. Loutan et al. [34] conducted a survey of 22 volunteer interpreters. Five of these interpreters (28%) reported frequent psychological difficulties while interpreting, and 12 (66%) reported frequent recall of painful memories. Previous research has shown that medical interpreters work in stressful healthcare-specific situations, such as illness, death, injury, treatment, and laboratory tests, and that they experience a lot of emotional distress when they empathize too much with patients [35,36]. In recent years, it has been proposed to strengthen the self-care of medical interpreters [37]. Knowledge and skills related to self-care to alleviate the psychological difficulties felt in interpreting may be useful in continuing medical interpreting.

### 4.1. Considering the Four Quadrants of Medical Communication in the Communication Skills of Medical Interpreters

We presented four quadrants divided by two axes (intervention and perspective) to illustrate the characteristics of the patient’s narrative. In the United States, the Bridging the Gap textbook recommends that medical interpreters should aim to convey messages without changing them, like a conduit, and that no intervention is performed [38]. The concept of incremental intervention was proposed in the Bridging the Gap textbook, which states that intervention may be performed depending on whether intervention is necessary in a particular situation, such as cultural mediation. However, the current findings indicated that active intervention can be implemented even in conventional medical care that does not require cultural mediation. According to the analysis of the interview results and the 2 × 2 grid, the ideal medical interpreter’s communication skills are considered to be active and fall somewhere between I and II.

Medical interpreters trained as intermediaries in communication increase the satisfaction of the relationship between the medical practitioner and the patient. In a study conducted by Baker et al., patients who felt that they needed an interpreter but did not have one reported lower satisfaction with their relationship with their healthcare provider, indicating that the provider was less friendly and less personally attentive, and that they were less comfortable than those who used an interpreter (88% of whom were ad hoc interpreters) [39].

There are growing expectations of professional medical interpreters, who utilize interpreting skills and medical knowledge to bridge the communication gap between patients and healthcare providers, and whose usefulness has been reported globally. Lindholm et al. and Abbato et al. noted that non-English-speaking patients who use medical interpreters had significantly shorter hospital stays [40,41].

### 4.2. Originality of This Research

In the current study, we conducted interviews and a survey to examine the communication skills of medical interpreters from the perspectives of both medical professionals and medical interpreters, and we compared the responses between these two groups.

In previous studies, interviews have been conducted with medical interpreters and collaboration between medical interpreters and medical professionals has been examined. However, it remains unclear whether there are differences in the ways medical practitioners and medical interpreters perceive the importance of various communication skills of medical interpreters.

In terms of originality, no previous study has interviewed both medical interpreters and healthcare professionals regarding the communication skills required of medical interpreters. We believe that this study has contributed to clarifying the role of medical interpreters, not only as language mediators but also as key communication facilitators between medical professionals and patients.

### 4.3. Limitations

Some medical professionals had experience as medical interpreters, and some medical interpreters had practical experience as medical practitioners. In addition, the sample included a mixture of individuals who practiced face-to-face interpretation and telephone interpretation, as well as inbound and volunteer interpretation. Therefore, the responses we collected may differ from the actual situation of some medical interpreters.

More research will be needed to determine how these differences affect medical interpreters’ perceptions of their communicative skills.

Interviews were conducted with medical interpreters and medical professionals. Because the respondents’ comments were based on recall, it is possible that they did not accurately capture the understanding of the experience at the time.

The reason patients and their families were not interviewed is that medical interpreters and healthcare professionals have extensive experience in a variety of medical interpretation situations. We believe that based on their clinical experience, they would have a clear understanding of the communication skills needed from medical interpreters. However, interviewing patients and their families would provide valuable insights, and we recognize this as an important area to explore in future research.

## 5. Conclusions

We explored medical professionals’ and medical interpreters’ perceptions regarding the communication skills of medical interpreters. Respondents’ narratives regarding the communication skills of medical interpreters were divided into two axes (intervention and perspective) and presented as a 2 × 2 grid with four quadrants.

The most important communication skills identified for medical interpreters included respecting others and understanding their opinions and positions, being attuned to the relationships around them and maintaining flexibility, understanding the dynamics between patients and healthcare professionals, and recognizing cultural and social differences such as lifestyle, values, health, medicine, and religion.

The key difference between the perspectives of medical interpreters and healthcare professionals was that medical professionals viewed interpreters as outsiders who, while not medical experts, were attuned to the patient’s thoughts and wishes. By contrast, medical interpreters did not perceive themselves as “outsiders” but saw themselves as part of the medical team, seeking to build a relationship of trust with the healthcare professionals.

## Figures and Tables

**Table 1:** Items of communication skills of medical interpreters.

**Number**	**Six Items of ENDCORES**
4b-1	Take control of your emotions and behaviors
4b-2	Express your thoughts and feelings well
4b-3	Correctly understand the thoughts and feelings that the other person wants to convey
4b-4	Ensure that your opinions and positions are accepted by others
4b-5	Respect others and understand their opinions and positions
4b-6	Work with the relationships around you and maintain flexibility
**Number**	**Ministry of Health, Labor and Welfare’s 5 Communication Skills of Medical Interpreters**
4b-7	Understand what communication is and understand the verbal and nonverbal messages that affect intercultural communication and communication
4b-8	Understand the relationship and communication between patients and healthcare professionals
4b-9	Understand the interpersonal communication skills required for medical interpretation and how to interact with patients
4b-10	Understand cultural and social differences such as lifestyle habits and values, religious views and ideas related to health, medical care, and communication
4b-11	Understand the cultural mediation of medical interpreters

**Table 2:** Basic attributes of interview respondents.

	A	B	C	D	E	F	G	H	I	J
	Interpreter	Interpreter	Interpreter	Interpreter	Interpreter	Nurse	Doctor	Nurse	Nurse/ Midwife	Doctor
Gender	Female	Female	Female	Female	Female	Female	Male	Female	Female	Female
Age	30s	50s	70s	50s	40s	20s	30s	30s	50s	30s
Years of interpretation/ hospital work	7	18	16	9	14	4	5	4	27	7
Work arrangements	Freelancer	Freelancer	Freelancer	Freelancer	Full-time employee	Part-time employee	Full-time employee	Full-time employee	Full-time employee	Part-time employee
Language	Chinese	English	English	Chinese	Chinese	English	English	Chinese	English	Chinese
Interpretation format	Telephone	In person	In person	In person	In person	In person	In person	In person	In person	In person
Language in which the healthcare consultation is carried out	Japanese	Japanese	Japanese	Japanese	Japanese	Japanese	English	Japanese	Japanese	Japanese
Language into which the interpreter interprets for the patients and their families	Chinese	English	English	Chinese	Chinese	English	English	Chinese	English	Chinese
The most important item for medical interpreters	4b-10	4b-5	4b-5	4b-6	4b-6	4b-8	4b-1	4b-3	4b-8	4b-10

## Data Availability

Data are contained within the article.

## References

[1] Immigration Services Agency of Japan. https://www.moj.go.jp/isa/.

[2] Nagata A., Hamai T., Kanda K. (2010). Risk of ad hoc interpreters on the medical care services for Brazilian residents in Japan. J. Int. Health.

[3] van Rosse F., de Bruijne M., Suurmond J., Essink-Bot M.L., Wagner C. (2016). Language barriers and patient safety risks in hospital care. A mixed methods study. Int. J. Nurs. Stud..

[4] International Society of Clinical Medicine. https://kokusairinshouigaku.jp/activities/authentication/m-interpreter/auth/auth01.html.

[5] Hamai T., Nagata A., Nishikawa H. (2017). The need for medical interpreters: A questionnaire survey of municipal hospitals in Japan. Nihon Koshu Eisei Zasshi (Jpn. Soc. Public Health).

[6] Ministry of Health, Labour and Welfare. https://www.mhlw.go.jp/content/10800000/001282378.pdf.

[7] Jacobs E.A., Lauderdale D.S., Meltzer D., Shorey J.M., Levinson W., Thisted R.A. (2001). Impact of interpreter services on delivery of health care to limited-English-proficient patients. J. Gen. Intern. Med..

[8] Harmsen H., Meeuwesen L., van Wieringen J., Bernsen R., Bruijnzeels M. (2003). When cultures meet in general practice: Intercultural differences between GPs and parents of child patients. Patient Educ. Couns..

[9] Lion K.C., Gritton J., Scannell J., Brown J.C., Ebel B.E., Klein E.J., Mangione-Smith R. (2021). Patterns and predictors of professional interpreter use in the pediatric emergency department. Pediatrics.

[10] Ministry of Health, Labour and Welfare of Japan. https://www.mhlw.go.jp/content/10800000/000856701.pdf.

[11] Fujimoto M., Daibo I. (2007). ENDCORE: A hierarchical structure theory of communication skills. Jpn. J. Personal..

[12] Nagatani Y. (2013). A Study of Professionalism of Dental Hygienist in Japan. Master’s Thesis.

[13] Otani T. (2011). SCAT: Steps for Coding and Theorization—A qualitative data analysis method that is easy to start with explicit procedures and can be applied to small-scale data. J. Jpn. Soc. Kansei Eng..

[14] Muramatsu N., Akiyama H. (2011). Japan: Super-aging society preparing for the future. Gerontologist.

[15] Japan Dental Hygienists’ Association. https://www.jdha.or.jp/english/index.html.

[16] Van de Camp K., Vernooij-Dassen M.J., Grol R.P., Bottema B.J. (2004). How to conceptualize professionalism: A qualitative study. Med. Teach..

[17] Otani T. (2007). “SCAT” a qualitative analysis method by four-step coding: Easy startable and small scale data applicable process of theorization. Bull. Grad. Sch. Educ. Hum. Dev. Educ. Sci. Nagoya Univ..

[18] Al-Rumayyan A., Van Mook W.N.K.A., Magzoub M.E., Al-Eraky M.M., Ferwana M., Khan M.A., Dolmans D. (2017). Medical professionalism frameworks across non-Western cultures: A narrative overview. Med. Teach..

[19] Japanese Nursing Association. https://www.nurse.or.jp/jna/english/activities/pdf/ethics2003.pdf.

[20] International Council of Nurses. https://www.icn.ch/.

[21] Japan Automobile Federation (JAF). https://jaf.or.jp/common/safety-drive/online-training/risk-prediction.

[22] Wadensjö C. (1998). Interpreting as Interaction.

[23] Bernardi E., Gnani F. (2022). The impact of the COVID-19 pandemic on medical interpreters/cultural mediators in Italy. FITISPos Int. J..

[24] Downey L.V., Zun L.S. (2010). The correlation between patient comprehension of their reason for hospital admission and overall patient satisfaction in the emergency department. J. Natl. Med. Assoc..

[25] Street R.L., Makoul G., Arora N.K., Epstein R.M. (2009). How does communication heal? Pathways linking clinician–patient communication to health outcomes. Patient Educ. Couns..

[26] Mayo R., Parker V.G., Sherrill W.W., Coltman K., Hudson M.F., Nichols C.M., Yates A.M., Pribonic A.P. (2016). Cutting corners: Provider perceptions of interpretation services and factors related to use of an ad hoc interpreter. Hisp. Health Care Int..

[27] Volpe R.L., Hopkins M., Geathers J., Watts Smith C., Cuffee Y. (2021). Negotiating professional identity formation in medicine as an “outsider”: The experience of professionalization for minoritized medical students. Qual. Res. Health.

[28] Köksala O., Yürük N. (2020). The role of translator in intercultural communication. Int. J. Curric. Instr..

[29] Pokorn N.K., Južnič T.M. (2020). Community interpreters versus intercultural mediators Is it really all about ethics?. Transl. Interpret. Stud..

[30] Sturman N., Farley R., Claudio F., Avila P. (2018). Improving the effectiveness of interpreted consultations: Australian interpreter, general practitioner and patient perspectives. Health Soc. Care Community.

[31] Williams A., Oulton K., Sell D., Wray J. (2018). Healthcare professional and interpreter perspectives on working with and caring for non-English speaking families in a tertiary paediatric healthcare setting. Ethn. Health.

[32] Kotovicz F., Getzin A., Vo T. (2018). Challenges of refugee health care: Perspectives of medical interpreters, case managers, and pharmacists. J. Patient Cent. Res. Rev..

[33] Ridgeway J.L., Njeru J.W., Breitkopf C.R., Mohamed A.A., Quirindongo-Cedeño O., Sia I.G., Wieland M.L. (2021). Closing the gap: Participatory formative evaluation to reduce cancer screening disparities among patients with limited English proficiency. J. Cancer Educ..

[34] Loutan L., Farinelli T., Pampallona S. (1999). Medical interpreters have feelings too. Soz. Praventivmed.

[35] Mizuno M., Naito M. (2015). Community Interpreting—Communication in a Multicultural Society.

[36] Rajpoot A., Rehman S., Ali P. (2020). Emotional and psychological impact of interpreting for clients with traumatic histories on interpreters: A review of qualitative articles. WikiJ Med..

[37] Oshimi T. (2019). The development of certification for health care interpreters in Japan. J. Int. Soc. Clin. Med..

[38] Cross Cultural Health Care Program (2014). Bridging the Gap: A Basic Textbook for Medical Interpreters: 40 Hours for Multilingual Interpreter Groups: Interpreter’s Handbook.

[39] Baker D.W., Hayes R., Fortier J.P. (1998). Interpreter use and satisfaction with interpersonal aspects of care for Spanish-speaking patients. Med. Care.

[40] Lindholm M., Hargraves J.L., Ferguson W.J., Reed G. (2012). Professional language interpretation and inpatient length of stay and readmission rates. J. Gen. Intern. Med..

[41] Abbato S., Greer R., Ryan J., Vayne-Bossert P., Good P. (2019). The impact of provision of professional language interpretation on length of stay and readmission rates in an acute care hospital setting. J. Immigr. Minor. Health.

